# Making connections — strategies for single molecule fluorescence biophysics

**DOI:** 10.1016/j.cbpa.2013.05.020

**Published:** 2013-08

**Authors:** Dina Grohmann, Finn Werner, Philip Tinnefeld

**Affiliations:** 1 Physikalische und Theoretische Chemie – NanoBioSciences, Technische Universität Braunschweig, Hans-Sommer-Strasse 10, 38106 Braunschweig, Germany; 2 RNAP Laboratory, Institute of Structural and Molecular Biology, Division of Biosciences, University College London, Darwin Building, Gower Street, London WC1E 6BT, United Kingdom

## Abstract

Fluorescence spectroscopy and fluorescence microscopy carried out on the single molecule level are elegant methods to decipher complex biological systems; it can provide a wealth of information that frequently is obscured in the averaging of ensemble measurements. Fluorescence can be used to localise a molecule, study its binding with interaction partners and ligands, or to follow conformational changes in large multicomponent systems. Efficient labelling of proteins and nucleic acids is very important for any fluorescence method, and equally the development of novel fluorophores has been crucial in making biomolecules amenable to single molecule fluorescence methods. In this paper we review novel coupling strategies that permit site-specific and efficient labelling of proteins. Furthermore, we will discuss progressive single molecule approaches that allow the detection of individual molecules and biomolecular complexes even directly isolated from cellular extracts at much higher and much lower concentrations than has been possible so far.

## Highlights

• The single-molecule approach yields exciting insights for many biomolecular applications.
• There are significant challenges to achieve main-stream single-molecule measurements.
• New labelling chemistries enable multiple tagged molecules *in vitro* and in live cells.
• Single-molecule pull-down expands the toolbox complementing co-immunoprecipitation.
• Breaking the single-molecule concentration barrier is within reach.

## Graphical abstract

**Figure:**
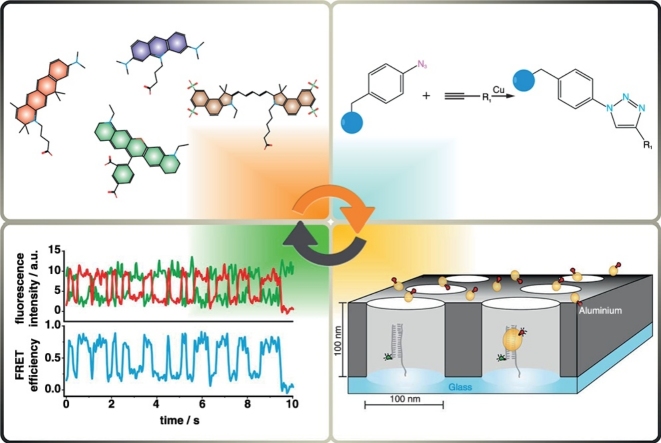

**Current Opinion in Chemical Biology** 2013, **17**:691–698

This review comes from a themed issue on **Molecular imaging**

Edited by **James Chen** and **Kazuya Kikuchi**

For a complete overview see the Issue and the Editorial

Available online 13th June 2013

1367-5931/$ – see front matter, © 2013 Elsevier Ltd. All rights reserved.

**http://dx.doi.org/10.1016/j.cbpa.2013.05.020**

## Introduction

Biophysical techniques have been invaluable to gain a detailed understanding of biological systems often providing quantitative and time-resolved data that complement data obtained by traditional biochemical experimental setups. Especially single molecule techniques like atomic force spectroscopy (AFM), magnetic and optical tweezers, fluorescence correlation spectroscopy (FCS) and single-molecule fluorescence spectroscopy provide exceptionally rich datasets that combine structural information with high time resolution [1^•^,2,3]. Because single molecule techniques avoid the averaging effect seen in bulk experiments, subpopulations, competing reaction pathways and transient intermediates can be identified. A fluorescent molecule is a highly sensitive molecular probe rich in information and sensitive to its environment. Among the measurable parameters are the spectral properties of the fluorophore (absorption and emission), the fluorescence intensity (‘brightness’), the quantum yield, the fluorescence lifetime and anisotropy. The use of two fluorophores in Förster resonance energy transfer (FRET) measurements [4–6] extends this set of variables to include the stoichiometry between the probes in the complex, their interaction with each other and the distance between them. All of these parameters can be obtained individually or in combination via multiparameter fluorescence detection [7–9]. Thereby, single molecule fluorescence measurements provide a wealth of information that inform directly about the status of a molecule. Still, many experiments cannot be carried out at the level of single molecules as many obstacles remain. Here, we review the recent advances to develop minimally invasive labelling schemes, to measure under physiological relevant conditions and to expand the range of concentrations suitable for single molecule measurements.

## *In vitro* fluorescence labelling

Of paramount importance for successful single molecule experiments is the quantitative and site-specific modification of molecules with fluorescent probes. For biological applications, a fluorescent label is ideally a small and water-soluble molecule in order to avoid aggregation and to prevent non-specific interactions with the biomolecule via hydrophobic interactions. The label has to be available in a form that it can be attached with high specificity to a protein side chain. Fluorescent dyes used for single molecule fluorescence applications commonly exhibit a maximum extinction coefficient *ɛ*_max_ > 80.000 mol^−1^ cm^−1^ and a fluorescence quantum yield of *Φ* > 0.1. Their fluorescence lifetime is of the order of a few nanoseconds and their size is roughly one nanometer.

Bioconjugation is commonly carried out with fluorophore derivatives that target the functional side chains of specific native or engineered amino acids in a protein. The fluorophore attachment site has to be carefully chosen in order to prevent label-induced alteration of the protein's activity and folding. The coupling reaction should be efficient in aqueous buffers at neutral pH and ambient temperatures as most proteins are not soluble in organic solvents and tend to unfold or aggregate at high temperatures and in highly basic or acidic environments. In addition, the coupling reaction needs to be highly chemoselective to ensure site-specific labelling of a single site in the protein. To this end coupling to amines and thiols are the most common labelling strategies that work efficiently under mild reaction conditions [10].

**Figure 1:**
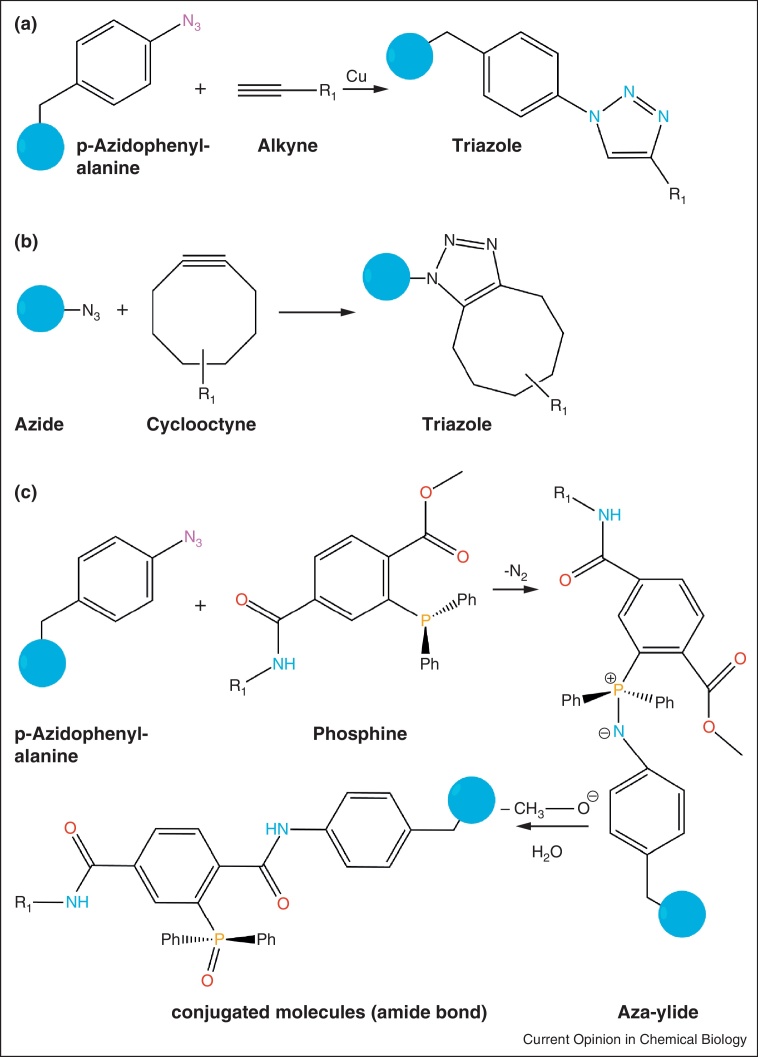
Protein labelling schemes with bioorthogonal ligation reactions. Three key bioorthogonal ligation reactions commonly used: **(a)** Cu(I) catalysed 3 + 2 cycloaddition reaction between an alkyne and an azide; **(b)** strain-promoted 3 + 2 cycloaddition reaction between a cyclooctyne and an azide and a **(c)** Staudinger–Bertozzi ligation between a phosphine and an organic azide. R1 denotes the side chain to which the fluorophore is coupled and the blue sphere symbolises the protein.

Newly developed technologies like bioorthogonal chemistry in combination with genetic engineering facilitate the site-specific labelling of unnatural amino acids (UAA) at any given position in a protein [11] improving the freedom of label positioning particularly in large proteins hitherto inaccessible for site-specific labelling because of first, their high cysteine content, second, an unfavourable position of the cysteine residue in the core of the protein or third, the essential role of the cysteine in the coordination of bivalent metal ions as seen in zinc-containing proteins. The coupling chemistries used in bioorthogonal reactions rely on unique chemical groups (e.g. para-acetyl or para-azide moieties) that are not part of the biological repertoire of amino acids [12^•^,13]. However, several conditions have to be fulfilled to make such a strategy successful. The UAA — that is supplied to the growth media — has to cross the membrane of the bacteria and be compatible with the bacterial metabolism (i.e. not be cytotoxic). A unique amber stop codon (TAG) is engineered into the desired labelling site that serves as a coding codon for the unnatural amino acid. Plasmid-borne pairs of engineered orthogonal tRNAs and aminoacyl-tRNA synthetases facilitate the efficient loading of the UAA to the tRNA and subsequent incorporation of the UAA at amber stop codons. tRNA loading by the tRNA synthetase has to be highly specific for the exogenous amino acid but at the same time needs to be compatible with the bacterial translation machinery. Directed protein evolution schemes yielded several orthogonal pairs that have been adapted for use in *Escherichia coli* [14–16]. Since the bacterial release factor RF-1 that recognizes amber stop codons is essential for bacterial cell viability, termination of translation at the derivatisation site competes with incorporation of the UAA and the incorporation efficiency does not exceed 20–30% [17]. The Staudinger–Bertozzi ligation between an azide and a triarylphosphine moiety (Figure 1c) and alternatively the copper-catalysed [3 + 2] cycloaddition between an azide and an alkyne group [18] (Figure 1a, also referred to as ‘click reaction’) are the most popular types of bioorthogonal reactions that can be used *in vitro* as well as *in vivo* because of their superior selectivity and biocompatibility [19]. Recently, copper-free click chemistry has emerged that relies on strain-promoted cycloaddition making the reaction suitable for *in vivo* applications and work with highly sensitive protein samples (Figure 1b) [20]. In parallel, UAAs have been developed that can serve as reactant in a copper-free cycloaddition [21]. Plass *et al.* demonstrated that this approach leads to fluorescently labelled proteins suitable for single molecule studies [22^•^]. The Staudinger–Bertozzi ligation and cycloaddition can also be employed if the UAA carries the alkyne and the fluorophore is modified with the azide group, which is an attractive option because azides are often reduction-sensitive and labile during biochemical purification [23].

Many single molecule studies are designed to address the conformational flexibility of proteins in solution, or the structural organization either of single proteins or protein complexes. Donor and acceptor probes for an intermolecular FRET system can be engineered into individual subunits that constitute a complex molecular machine or a heteromeric complex following standard coupling chemistries. In contrast, site-specific incorporation of donor and acceptor fluorophore in a single polypeptide is challenging and requires multiple unique coupling sites for differential labelling. A combination of the described coupling techniques often lead to successful dual labelling. For example, the N-terminus of a protein can be labelled via an amine-reactive group and a single cysteine with a thiol-reactive group. Likewise, the modification of a single cysteine and an unnatural amino acid in a single protein chain is a sensible approach for an intramolecular site-specific labelling [23]. The incorporation of multiple [24] and two different UAAs [25] has been described, which opens the door for stochastic and site-specific labelling of proteins via the reactive side-chains of the UAAs. In some cases the site-specific positioning of the donor or acceptor probe is not mandatory to analyse the conformational flexibility or folding of a protein. Here, labelling via identical reactive moieties (e.g. 2 cysteines or 2 UAAs) is practicable [26]. Recently, advanced labelling strategies have been utilized to allow even triple-colour labelling within a single protein (stochastical labelling of two cysteines and one UAA) [27].

## Performing single-molecule experiments preserving biological relevant interactions

**Figure 2:**
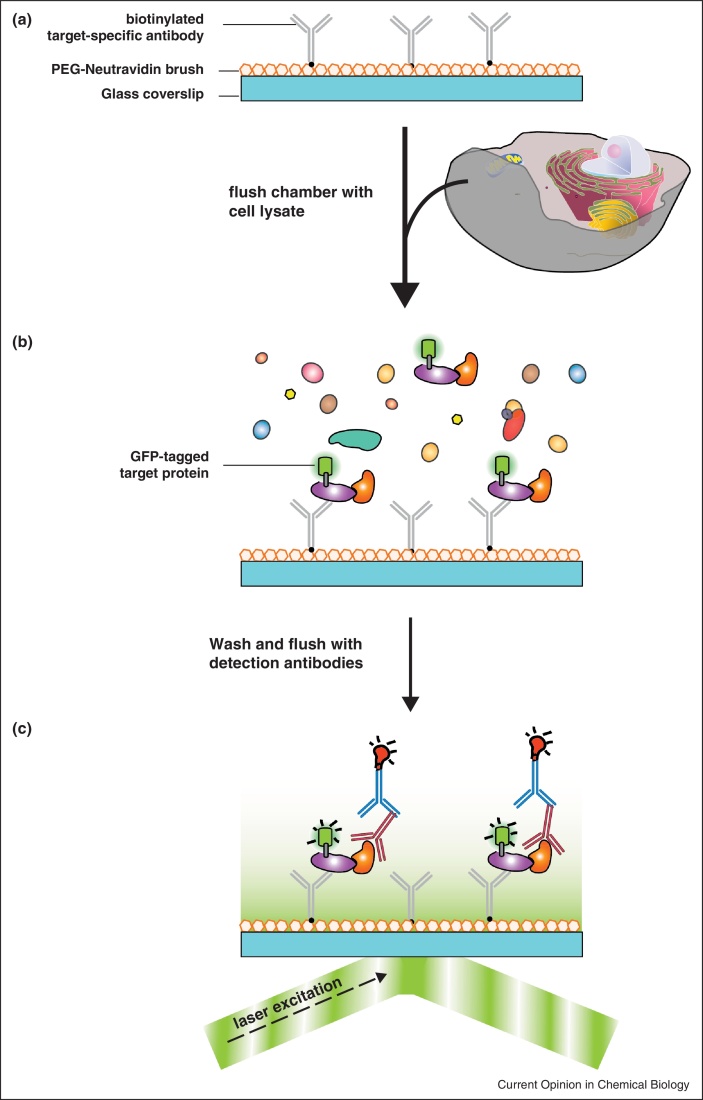
Workflow of a single-molecule pull-down (SiMPull) experiment [28^••^]. In a single-molecule immunoprecipitation experiment antibodies directed against the protein of interest are directly immobilised via a biotin–neutravidin interaction on the imaging surface for single molecule experiments first **(a)**. Subsequently, the cell lysate is flushed into the measuring chamber and the target protein is captured by the antibody **(b)**. After removal of the unbound molecules a second set of antibodies is used to identify interaction partners (bait protein) of the target protein **(c)**. Using a total internal reflection microscope target and bait proteins are visualised either by the emission of a fluorescent protein like GFP fused to one of the proteins or by the fluorescence signal of a dye-labelled antibody.

In most cases single molecule fluorescence measurements are performed using *in vitro* labelled recombinant proteins. However, in many instances the protein of interest cannot be obtained from heterologous expression and consequently efficient *in vitro* labelling becomes a challenging task. From a biological perspective, proteins do not act individually but are often part of a complex interaction network that is regulated in space and time. Hence, an investigation of isolated molecules is revealing just one layer of information but does not reflect the complex scenario found in a cellular environment. These drawbacks can be overcome employing the recently developed single-molecule pull-down [28^••^]. This approach extends the well-known co-immunoprecipitation technique to the single-molecule level (termed SiMPull). A target protein is directly captured from the cell lysate using a specific antibody or protein tag. At the same time interaction partners are co-purified. A subsequent washing step removes all unbound proteins and immobilised target proteins or protein complexes can be directly visualised using either a fluorescent fusion protein or the dye-labelled antibody (Figure 2). Sample preparation is quick and mild preserving biological conditions and increasing the probability to capture weak or transient interactions. The direct immobilisation of endogenous complexes from cellular extracts on a cover slip provides a wealth of information and informs for example about stoichiometries within the protein complex, the oligomerisation state of a protein, the expression level of a specific protein [30]. Furthermore, the catalytic activity of an enzyme can be directly monitored after extraction [28^••^]. The SiMPull technique has been employed to study the function of complex biomolecular machineries composed of multiple subunits like the eukaryotic spliceosome [31] and the replisome [32,33] but includes also studies on protein kinases and the mTOR signalling complex [28^••^] (for an overview see [34^•^]). SimPull opens up the possibility to visualise complex macromolecular machineries not amenable to *in vitro* assembly as they can be directly reconstituted on the cover slip (e.g. the eukaryotic replisome) [29] and the order of assembly can be entangled. The activity of the machinery can be monitored in the presence of cellular co-factors that have not been found to interact with the complex with conventional biochemical methods because of labile interactions. As single molecule fluorescence techniques are highly sensitive minimal amounts of the molecule of interest are sufficient for measurements. Hence, expression of a GFP-tagged target protein can be adjusted to endogenous levels minimising the disturbance of the finely tuned cellular network.

## Overcoming the concentration problem

**Figure 3:**
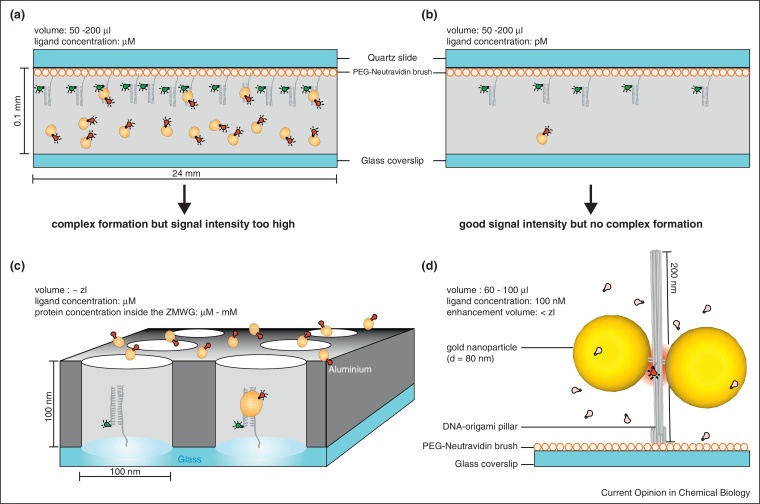
The concentration barrier in single molecule measurements exemplified by a colocalisation experiment. In order to detect fluorescence intensities from individual molecules the concentration of the fluorescently labelled species is usually reduced to picomolar concentrations **(b)**. In contrast, many biomolecular interactions are characterised by dissociation constants in the micromolar to millimolar range. Raising the concentration of the labelled biomolecule to allow complex formation leads to high fluorescence intensities that prevent measurements on individual molecules **(a)**. a and b depict a measurement chamber for total internal fluorescence (TIRF) microscopy. In order to access interactions with low or medium affinity the detection volume has to be reduced to zeptoliters as it has been realised using zeromode waveguides **(c)**. Even at micromolar to millimolar concentrations it is very likely that just one molecule occupies the volume at any time. **(d)** Alternatively, the fluorescence intensity of a single fluorophore under investigation positioned close to metallic nanoparticles [50^••^] is increased by at least two orders of magnitude. The fluorescent probe is positioned in a plasmonic hot-spot created in the centre of two nanoparticles. The accurate geometry of the self-assembled nanoantenna is achieved employing the DNA-origami technique. This situation is illustrated for immobilised molecules whose fluorescence is monitored by confocal microscopy. Compared to the signal intensity of the enhanced fluorophore the fluorescence intensities of the surrounding fluorophores (up to higher nanomolar concentration) vanish in the background. Fluorophores are symbolised by green and red lightbulbs.

For many non-specialists, single molecule techniques seem ‘sophisticated’. The question for the single molecule spectroscopist is rather why one should do an ensemble experiment if the problem can be addressed on the single molecule level. The single molecule approach seems like the more direct one that in addition avoids an underestimation of sample heterogeneity. In fact, single molecule experiments often do not require highly pure or high quality samples since the single molecule spectroscopic parameters can be used to sort molecules and to select subpopulations for further analysis that meet specified criteria. However, experiments have to be carefully thought through as concentration is a critical parameter in single molecule experimental approaches (Figure 3a and b). Because of the diffraction limited optics samples are diluted to the picomolar to lower nanomolar concentration range so that indeed only one molecule resides in the diffraction limited (∼femtomolar) observation volume. Therefore, weak interactions that are only significantly populated at micromolar concentrations cannot be visualised. This drawback applies to many enzyme substrate interactions since Michaelis–Menten constants are commonly found to be in the micromolar range [35]. On the other hand, very low concentrations (<femtomolar) can also not be detected since very dilute analytes do not diffuse through the observation volume in the time scale of the experiment. Dilute concentrations are of interests in analytics and diagnostics, for example, to detect pathogens or ultra-dilute marker molecules. The issue here is to efficiently detect these individual analytes among the background fluorescence. Increasing the detection volume does not solve the problem since the noise scales with the number of solvent and impurity molecules. These issues are the main reasons why commercial applications of single molecule detection have been limited. Interestingly, the two outstanding applications are single molecule sequencing and superresolution microscopy by subsequent single molecule localizations [36,37]. Both techniques distinguish themselves by overcoming the concentration limitations, although in very different ways.

In recent years, different approaches have been developed to overcome this concentration barrier. Molecules have been trapped in small surface-tethered lipid vesicles that have an approximately 100-fold smaller than diffraction-limited observation volume [38,39]. Photoactivatable probes in a microfluidic flow have been used to focus on the molecules that bound to the target molecules [40^•^] while other photoactivated molecules are washed away.

Nanophotonics offers solutions to the concentration range problem of single molecule detection by directly reducing the effective observation volume. It might become the central ingredient for further advancement although the size reduction and the high surface to volume ratio might also not be biocompatible in all cases. Circular holes of 50–200 nm diameter in a metal cladding film of 100 nm thickness deposited on a transparent substrate (so called zeromode waveguides), for example, reduce the observation volumes and enable monitoring of enzymatic reactions at high substrate concentration (Figure 3c) [41^••^]. This technique led to the visualization of DNA polymerization and translation at the single-molecule level [36,42]. However, the production and handling of these nanophotonics structures is costly and serial by nature. Since molecules are not specifically placed in the centre of the structures, they experience varying levels of fluorescence quenching due to the distribution of distances to the metallic walls yielding heterogeneous signals.

Instead of physically suppressing the light field around the fluorophore by means of metals, an alternative approach is to locally enhance fluorescence using optical antennas (Figure 3d) [43]. The interaction of metal nanostructures with fluorescent dyes is very complex and can involve fluorescence increase by increasing the local excitation field and the radiative rate of the fluorescent dye. On the other hand, fluorescence can also be quenched and the energy be absorbed by the metal nanostructures. More and more reports in recent years have indicated the specific requirements to achieve fluorescence enhancements of up to more than 1000-fold [44]. To exploit this approach for single-molecule assays a reproducible control of the enhancement hot-spots, for example, by the arrangements of noble metal nanoparticles is required. In addition, a handle is essential to place the single-molecule assay of interest in the hot-spot created by the nanoparticle.

We anticipate that DNA origami structures [45,46] can represent the scaffold to which not only nanoparticles but also docking sites for single-molecule assays can be attached. DNA origami are self-assembled 2D and 3D nanostructures based on the single-stranded DNA genome of bacteriophage M13 that is folded with the help of hundreds of short oligonucleotides called ‘staple strands’ [45]. Crucially, these nanoassemblies allow a spatially defined arrangement of functional entities like for example biotins, nanoparticles or docking strands for biomolecular assays [47–49]. This has recently been exploited in the form of DNA origami with the shape of a nanopillar [50^••^]. Nanoparticle dimers attached to the DNA origami act as an antenna and focus the light in their centre where a single-molecule assay might be attached by further protruding DNA strands. At a gap of 23 nm that might be sufficient to place, for example, an enzyme a fluorescence enhancement of up to 100-fold could be obtained. Since the created hot-spots are ultra-small the enhancement is restricted to the molecules in the hotspot and additional labelled species (even present at elevated concentrations) in the surrounding solution vanish compared to the increased signal in the hot-spot. This opens the possibility to solve the concentration issue and allow single molecule assays at elevated concentrations. Moreover, fluorescence enhancement in the hot-spot might also increase the signal-to-noise ratio to an extent that single molecules can even be detected in much bigger volumes opening the route to single molecule diagnostics at ultra-low concentrations.

## Conclusions

Single molecule fluorescence measurements provide valuable information about biomolecular mechanisms but there are a number of parameters that have to be checked when planning a single molecule experiment in order to access whether the biological process can be studied with single molecule techniques. These considerations mainly concern the concentration range, the time scale of the molecular process and the availability of efficiently labelled molecules of the system under investigation. Recent developments brought about optimised fluorescent labelling protocols that allow selective labelling of unique reactive moieties of UAAs even in cell extracts. A combination with the powerful SiMPull technique, where minimal amounts of labelled species are sufficient for a single molecule experiment, potentially allow for single cell investigations checking on, for example, protein levels in differently stimulated cells. Finally the progressive fluorescence enhancement approaches that allow the detection of individual molecules at much higher and much lower concentrations than has been possible so far extend the range of applications to diagnostics and transient biological interactions in a high-throughput format.

## References and recommended reading

Papers of particular interest, published within the period of review, have been highlighted as:

• of special interest
•• of outstanding interest
